# Flavocoxid, a Natural Antioxidant, Protects Mouse Kidney from Cadmium-Induced Toxicity

**DOI:** 10.1155/2018/9162946

**Published:** 2018-04-18

**Authors:** Antonio Micali, Giovanni Pallio, Natasha Irrera, Herbert Marini, Vincenzo Trichilo, Domenico Puzzolo, Antonina Pisani, Consuelo Malta, Giuseppe Santoro, Rosalba Laurà, Domenico Santoro, Francesco Squadrito, Domenica Altavilla, Antonino Germanà, Letteria Minutoli

**Affiliations:** ^1^Department of Biomedical and Dental Sciences and Morphofunctional Imaging, University of Messina, Messina, Italy; ^2^Department of Clinical and Experimental Medicine, University of Messina, Messina, Italy; ^3^Department of Veterinary Sciences, University of Messina, Messina, Italy

## Abstract

**Background:**

Cadmium (Cd), a diffused environmental pollutant, has adverse effects on urinary apparatus. The role of flavocoxid, a natural flavonoid with antioxidant activity, on the morphological and biochemical changes induced *in vivo* by Cd in mice kidney was evaluated.

**Methods:**

C57 BL/6J mice received 0.9% NaCl alone, flavocoxid (20 mg/kg/day i.p.) alone, Cd chloride (CdCl_2_) (2 mg/kg/day i.p.) alone, or CdCl_2_ plus flavocoxid (2 mg/kg/day i.p. plus 20 mg/kg/day i.p.) for 14 days. The kidneys were processed for biochemical, structural, ultrastructural, and morphometric evaluation.

**Results:**

Cd treatment alone significantly increased urea nitrogen and creatinine, iNOS, MMP-9, and pERK 1/2 expression and protein carbonyl; reduced GSH, GR, and GPx; and induced structural and ultrastructural changes in the glomeruli and in the tubular epithelium. After 14 days of treatment, flavocoxid administration reduced urea nitrogen and creatinine, iNOS, MMP-9, and pERK 1/2 expression and protein carbonyl; increased GSH, GR, and GPx; and showed an evident preservation of the glomerular and tubular structure and ultrastructure.

**Conclusions:**

A protective role of flavocoxid against Cd-induced oxidative damages in mouse kidney was demonstrated for the first time. Flavocoxid may have a promising antioxidant role against environmental Cd harmful effects on glomerular and tubular lesions.

## 1. Introduction

Cadmium (Cd) is an environmental and industrial pollutant with high toxicity and carcinogenic activity [1]. The exposure to Cd is progressively increasing, owing to the wide use of Cd-containing goods in industrialized countries and to its long biological half-life (10–30 years) [2]. Environmental Cd may accumulate in many organs, such as liver [3], lungs, particularly in smokers [4], testes [5], and bones [6], harmfully interfering with their functions.

However, the main target of Cd is considered the kidney [7, 8], where it accumulates, owing to the absence of a specific mechanism for elimination [9]. In fact, damaged liver cells release a Cd-metallothionein (MT) complex which is filtered from the glomerulus and then endocytosed by the cells of the proximal tubule, where it is degraded by lysosomes: in this way, free Cd is released in the tubules [10], where 99% of the filtered Cd is reabsorbed [11], resulting in accumulation and consequent nephrotoxicity. In particular, it was demonstrated that the target sites of Cd are the proximal tubules [12]; in these structures, lesions of the brush border and fragmentation of the epithelial cells with the granular cytoplasm were observed [13–17]. However, Cd-induced structural damages of the glomeruli [18], consisting in increased mesangial matrix, glomerular swelling, and increased urinary space, were also demonstrated [19].

The mechanisms of Cd renal toxicity seem to be correlated mainly to its oxidative property, depleting major cellular antioxidants, such as thiol-containing antioxidants, and different enzymes involved in the protection against oxidative stress. However, Cd, differently from other heavy metals, is unable to produce directly reactive oxygen species (ROS). In fact, Cd replaces iron and copper from many cellular proteins, thus increasing the concentration of these unbound ions. The latter induce oxidative stress via Fenton reactions [20]. As a consequence, ROS might trigger the production of signaling molecules and proinflammatory cytokines leading to renal tissue damage [9, 21, 22]. Further mechanisms of Cd renal toxicity have been also described: among them, mitochondrial damage [23], cellular death, in particular apoptosis, induction [24], disruption of cadherin-mediated cell-cell adhesion in the proximal tubule cells [25], and stimulation of the inflammation pathways [9] were observed.

In particular, current evidence suggests that in CdCl_2_-treated mice, an increased expression of inducible nitric oxide synthase (iNOS) occurred in renal tissue, which could be related to the structural lesions of tubular epithelial cells [26]. Similarly, the overproduction of ROS might activate many signaling protein kinases [27], among which an important role is played by extracellular signal-related kinases (ERKs) 1 and 2. Furthermore, in patients with resistant albuminuria [28], oxidative stress increased the activity of matrix metalloproteinase- (MMP-) 9, which has a great specificity for substrates such as different types of collagen, proteoglycans, and elastin, particularly in basement membranes [29].

Several therapeutic approaches were proposed to prevent structural and functional damages following environmental or experimental Cd exposure, with particular attention to the protective functions of natural antioxidants [30–34].

Among them, flavonoids, polyphenolic compounds widely distributed in dietary fruits, vegetables, and wine, were evaluated in the treatment of different diseases [35, 36]. In particular, flavocoxid, a flavonoid containing both baicalin, extracted from *Scutellaria baicalensis* (Chinese skullcap), and catechin, extracted from *Acacia catechu* (Black catechu), showed a strong and tough antioxidant activity [37] and demonstrated a protective role against Cd-induced damages of the blood-testis barrier, reducing testicular damage and germ cell impairment in mice [38].

Therefore, we performed a biochemical, morphological, and morphometric study in mice exposed to Cd with and without flavocoxid coadministration, in order to evaluate the role of this flavonoid on mouse kidney and to propose it as an antioxidant tool in the therapy of human nephrotoxicity induced by the exposition to environmental Cd.

## 2. Materials and Methods

### 2.1. Experimental Protocol

All procedures complied with the standards stated in the Guide for the Care and Use of Laboratory Animals (Institute of Laboratory Animal Resources, National Academy of Sciences, Bethesda, Maryland). Forty-eight male C57 BL/6J mice (25–30 g) were purchased from Charles River Laboratories Italia srl (Calco, LC, Italy). The animals were provided a standard diet ad libitum with free access to tap water and were maintained on a 12-hour light/dark cycle. The animals were divided into four groups to receive vehicle (0.9% NaCl) alone, flavocoxid alone (20 mg/kg/day i.p.), cadmium chloride (CdCl_2_, 2 mg/kg/day i.p.) alone [38], or CdCl_2_ (2 mg/kg/day i.p.) plus flavocoxid (20 mg/kg/day i.p.), respectively. CdCl_2_ was dissolved in 0.9% NaCl. Seven animals/group were processed for biochemical and structural analysis; five animals/group were used for ultrastructural analysis. All mice were sacrificed after 14 days of treatment with an i.p. overdose of ketamine and xylazine, and bilateral nephrectomies were performed.

### 2.2. Serum Analysis

Blood samples were collected, left for 60 minutes to clot, and centrifuged for 15 minutes at 6000 rpm. Urea nitrogen was measured with a colorimetric kit strictly following the manufacturer's recommendations (Roche Diagnostics GmbH, Germany). Creatinine levels were measured with an enzymatic assay method using an automatic analyzer (Modular Roche Diagnostics GmbH, Germany).

### 2.3. Determination of Protein Content

Total cellular proteins were extracted in a lysis buffer composed of 25 mM Tris-HCl pH 7.4, 1.0 mM ethylene glycol tetraacetic acid (EGTA), 1.0 mM ethylenediaminetetraacetic acid (EDTA), and 0.5 mM phenylmethylsulphonyl fluoride, added with protease and phosphatase inhibitors [100 mM Na_3_VO_4_, aprotinin, leupeptin, and pepstatin (10 *μ*g/ml each)]. The cell lysate was centrifugated at 13000 rpm for 15 minutes, and the supernatant was used for protein concentration determination by Bio-Rad protein assay (Bio-Rad, Richmond, CA, USA).

### 2.4. Determination of Protein Carbonyls and Glutathione (GSH) Content

Total protein carbonyl content was determined in the kidney of all experimental groups with the DNPH assay, as described in detail by Gong et al. [39], and expressed in *μ*mol/mg protein. GSH content (nonprotein sulphydryl content) was also determined in the kidneys of all experimental groups according to the method of Ellman [40], as proposed by Gong [39].

### 2.5. Determination of Antioxidant Enzyme Content

Glutathione reductase (GR) activity was evaluated following the method of Smith et al. [41], while glutathione peroxidase (GPx) was determined according to Flohe and Gunzler [42], both described in detail by Manna et al. [43].

### 2.6. Determination of iNOS, pERK 1/2, and MMP-9 by Western Blot Analysis

The supernatant was diluted with Laemmli buffer (Sigma-Aldrich Srl, Milan, Italy). Protein samples were denatured in reducing buffer (62 mM Tris, pH 6.8, 10% glycerol, 2% SDS, 5% *β*-mercaptoethanol, and 0.003% bromophenol blue) and separated by electrophoresis on SDS polyacrylamide gel (6% or 10%), approximately for 1 h. The separated proteins were transferred to a PVDF membrane in a transfer buffer [39 mM glycine, 48 mM Tris-HCl (pH 8.3), and 20% methanol] at 200 mA for 1 h. The membranes were then blocked with 5% nonfat dry milk in TBS-0.1% Tween-20 for 1 h at room temperature. Membranes were washed three times for 10 min each in TBS-0.1% Tween-20 and incubated with a primary antibody for iNOS, pERK 1/2, and MMP-9 (Cell Signaling, Beverly, MA, USA) diluted in TBS-0.1% Tween-20 overnight at 4°C. The day after, the membranes were washed three times for 10 min in TBS-0.1% Tween-20 and were incubated with a specific peroxidase-conjugated secondary antibody (KPL, USA) for 1 h at room temperature. Following other washings, the membranes were analyzed by enhanced chemiluminescence (KPL, USA). Protein signals were quantified by scanning densitometry using a bioimage analysis system (C-DiGit Blot Scanner with Image Studio 4.0 software, LI-COR, Lincoln, Nebraska, USA), and the results were expressed as relative integrated intensity compared to controls. *β*-Actin (Cell Signaling Technology, Beverly, MA, USA) was used to confirm equal protein loading and blotting.

### 2.7. Histological Evaluation

The kidneys were fixed in 4% paraformaldehyde in 0.2 M phosphate-buffered saline (PBS), dehydrated in graded ethanol, cleared in xylene, and embedded in paraffin (Paraplast, SPI Supplies, West Chester, PA, USA). 5 *μ*m sections were stained with hematoxylin and eosin (HE), periodic acid-Schiff (PAS), and Sirius Red (SR). The slides were photographed with a Nikon Ci-L (Nikon Instruments, Tokyo, Japan) light microscope; the images were taken with a digital camera Nikon DS-Ri2.

### 2.8. Morphometric Evaluation

All quantitative evaluations were performed independently by two blind investigators (DP and AM). Images of twenty glomeruli from the cortical region obtained from ten HE-stained nonserial sections of each group were analyzed using the ImageJ software (National Institutes of Health, Bethesda, MD, USA) to determine the mean total glomerular area (TGA) [44].

Tubular damage was evaluated from twenty microscopic fields (800x) obtained from PAS-stained nonserial sections of each group according to the following arbitrary score: 0 = no damage; 0.5 = thinning of the brush border with or without interstitial edema; 1 = thinning of the tubular epithelia with or without interstitial edema; 2 = partial absence of the tubular epithelium with or without interstitial edema; and 3 = tubular necrosis with or without interstitial edema [45, 46].

For the assessment of the renal fibrosis, a quantitative evaluation of micrographs taken from twenty microscopic fields (800x) obtained from SR-stained nonserial sections of each group was performed by the Adobe Photoshop CS5 12.1 software, recording the pink/red color of collagen fibers. Positive areas were automatically estimated on the basis of their pixel number. Data were expressed as pixel number of positive-stained area/unit area (UA), considered as the entire micrograph area.

### 2.9. Transmission Electron Microscopy (TEM)

The kidneys of five mice from each group were fixed by immersion in 2.5% glutaraldehyde in 0.1 M phosphate buffer (pH 7.4) at +4°C, washed with 0.1 M phosphate buffer (pH 7.4), and postfixed in 1% OsO_4_ in 0.2 M phosphate buffer (pH 7.4) at +4°C for 1 h. The specimens were then dehydrated in graded ethanol, immersed in propylene oxide, and embedded in Durcupan (Sigma-Aldrich/Fluka, St. Louis, MO). After control with semithin sections, ultrathin sections were cut with a diamond knife on a Reichert-Jung Ultracut E, collected on uncoated 200 mesh copper grids, contrasted with methanolic uranyl acetate and lead citrate [47], and photographed with a JEOL-JEM-100 SX transmission electron microscope at 80 kV.

### 2.10. Drugs and Chemicals

CdCl_2_ was purchased from Sigma-Aldrich Srl (Milan, Italy). Flavocoxid (Limbrel®) was a kind gift of Primus Pharmaceuticals, Inc. (Scottsdale, Arizona, USA). All chemicals not otherwise mentioned were commercially available reagent grade.

### 2.11. Statistical Analysis

Values are provided as mean ± standard error (SE). The statistical significance of differences between group mean values was established using the Student's *t*-test. The statistical evaluation of differences among groups was performed with ANOVA comparison tests. Mann–Whitney *U* tests with Bonferroni correction were used for the statistical analysis of histological scores. A *p* value ≤ 0.05 was considered statistically significant.

## 3. Results

### 3.1. Flavocoxid Effects on Urea Nitrogen and Creatinine

Mice challenged with CdCl_2_ showed significant increases in urea nitrogen and creatinine levels when compared to both control groups. On the contrary, a significant reduction in urea nitrogen and creatinine was observed in CdCl_2_-challenged animals cotreated with flavocoxid (Table 1).

### 3.2. Flavocoxid Effects on Protein Carbonyls and GSH Content

The levels of protein carbonyl contents were significantly increased in Cd-challenged mice. The coadministration of CdCl_2_ and flavocoxid significantly decreased the levels of protein carbonyls in kidney (Table 2). On the contrary, a significant decrease in the activity of GSH was observed in Cd-challenged mice. The treatment with flavocoxid significantly increased GSH levels in kidneys of Cd-treated mice (Table 2).

### 3.3. Flavocoxid Effects on Antioxidant Enzyme Content

As a consequence of oxidative stress, a significant decrease in GR and GPx levels was observed in CdCl_2_-challenged mice. The coadministration with flavocoxid significantly increased the levels of antioxidant enzymes in the kidneys of Cd-treated mice (Table 3).

### 3.4. Flavocoxid Effects on iNOS, pERK 1/2, and MMP-9 Expression

**Figure 1:**
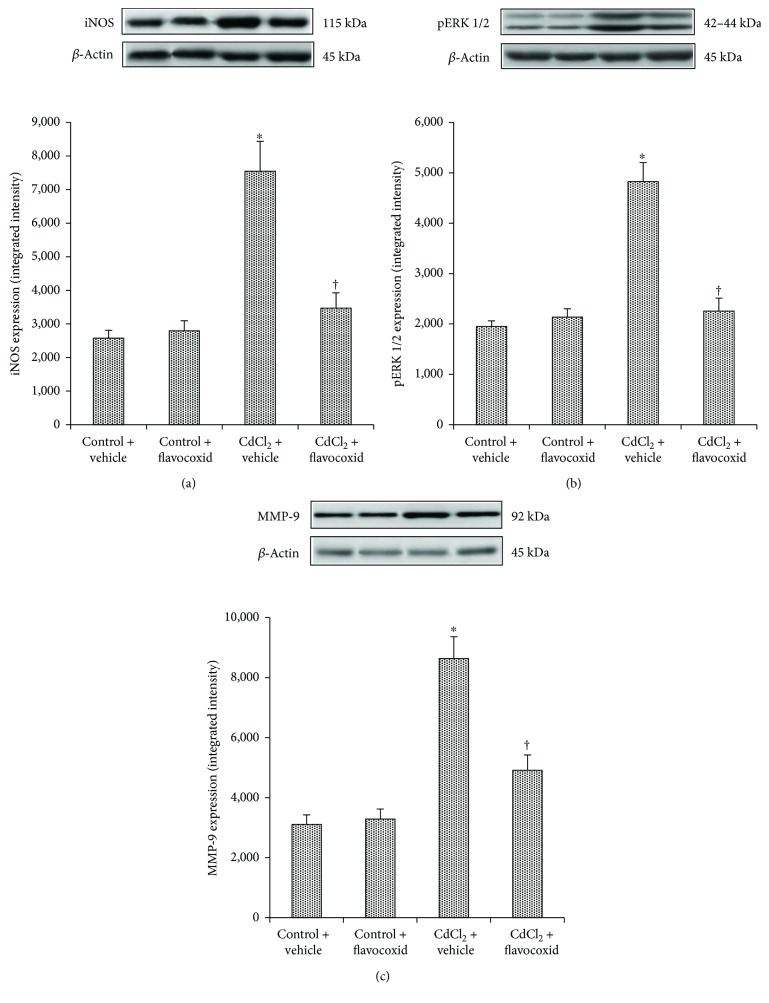
Representative Western blot analysis of iNOS (a), pERK 1/2 (b), and MMP-9 (c) of kidneys from mice of control plus vehicle (0.9% NaCl), control plus flavocoxid (20 mg/kg/day i.p.), CdCl_2_ (2 mg/kg/day i.p.) plus vehicle, and CdCl_2_ plus flavocoxid groups. ^∗^*p* < 0.05 versus both controls and ^†^*p* < 0.05 versus CdCl_2_ plus vehicle. Bars represent the mean ± SE of seven experiments.

A low and not statistically significant different expression of iNOS was detected in the kidneys of all control animals treated for 14 days with vehicle alone or flavocoxid alone (20 mg/kg/day i.p.). CdCl_2_ challenge induced a significant increase in iNOS expression after 14 days of administration. In CdCl_2_-challenged animals cotreated with flavocoxid, iNOS expression was significantly reduced (Figure 1(a)).

In all control animals treated for 14 days with vehicle alone or flavocoxid alone (20 mg/kg/day i.p.), pERK 1/2 expression was not statistically different. Conversely, pERK 1/2 expression was significantly increased after 14 days of CdCl_2_ administration. The coadministration of CdCl_2_ and flavocoxid significantly reduced pERK 1/2 expression (Figure 1(b)).

In the kidneys of all control animals treated for 14 days with vehicle alone or flavocoxid alone (20 mg/kg/day i.p.), MMP-9 expression was low and not statistically significantly different. MMP-9 expression was significantly increased after 14 days of CdCl_2_ administration, while it was significantly reduced after treatment with flavocoxid (Figure 1(c)).

### 3.5. Histological and Morphometric Evaluations

**Figure 2:**
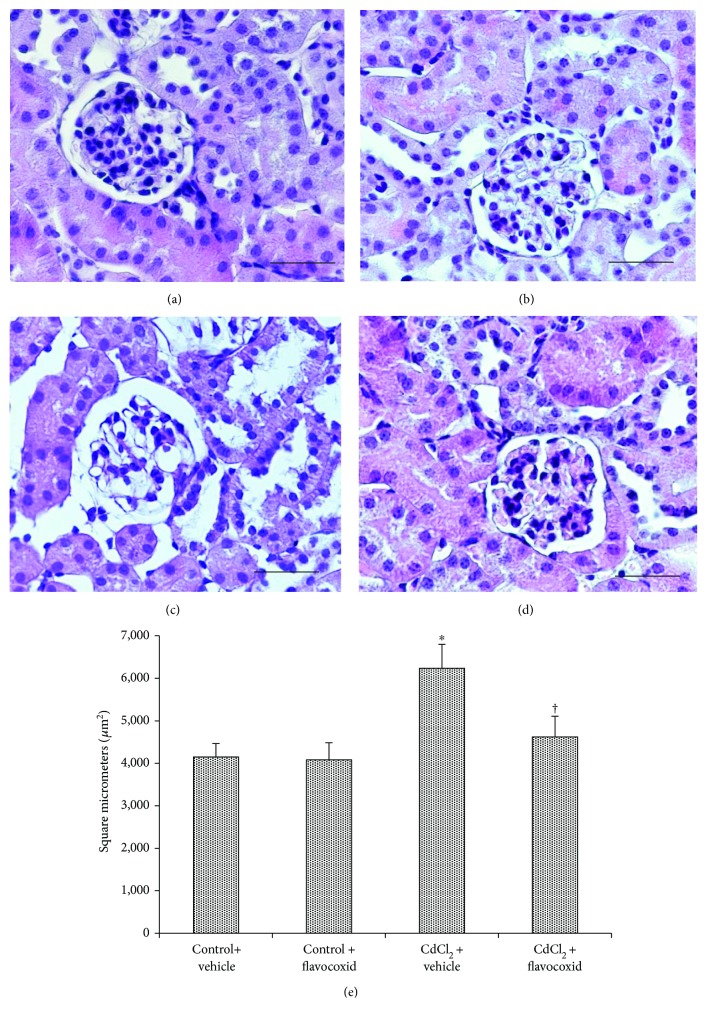
Structural organization of glomeruli and tubules of kidneys from mice of control plus vehicle (0.9% NaCl, 1 ml/kg/day i.p.), control plus flavocoxid (20 mg/kg/day i.p.), CdCl_2_ (2 mg/kg/day i.p.) plus vehicle, and CdCl_2_ plus flavocoxid groups (hematoxylin and eosin stain). (a, b) In both control groups, glomeruli and tubules show normal architecture. (c) In CdCl_2_-challenged mice, glomeruli present enlarged Bowman's space (arrow), while tubules show epithelial lesions (arrowhead). A mild interstitial edema is also present (∗). (d) In CdCl_2_-challenged mice administered with flavocoxid, glomerular and tubular morphology is normal. (e) Glomerular area evaluation. ^∗^*p* < 0.05 versus both controls and ^†^*p* < 0.05 versus CdCl_2_ plus vehicle (scale bar: 50 *μ*m).

For histological evaluation, kidney sections stained with HE, PAS, and Sirius red were examined. In kidney sections stained with HE of both control groups of mice, glomeruli and tubules had a normal histological structure (Figures 2(a) and 2(b)). In CdCl_2_-challenged mice, glomeruli showed enlarged Bowman's space, proximal tubules evidenced epithelial damages, and a mild interstitial edema was present (Figure 2(c)). In CdCl_2_-challenged mice administered with flavocoxid, glomerular and tubular morphology was normal (Figure 2(d)). The glomerular area morphometry demonstrated a significantly higher score in CdCl_2_-challenged mice, while in the CdCl_2_ plus flavocoxid group, the score was similar to controls and statistically significantly lower versus the CdCl_2_ group (Figure 2(e)).

**Figure 3:**
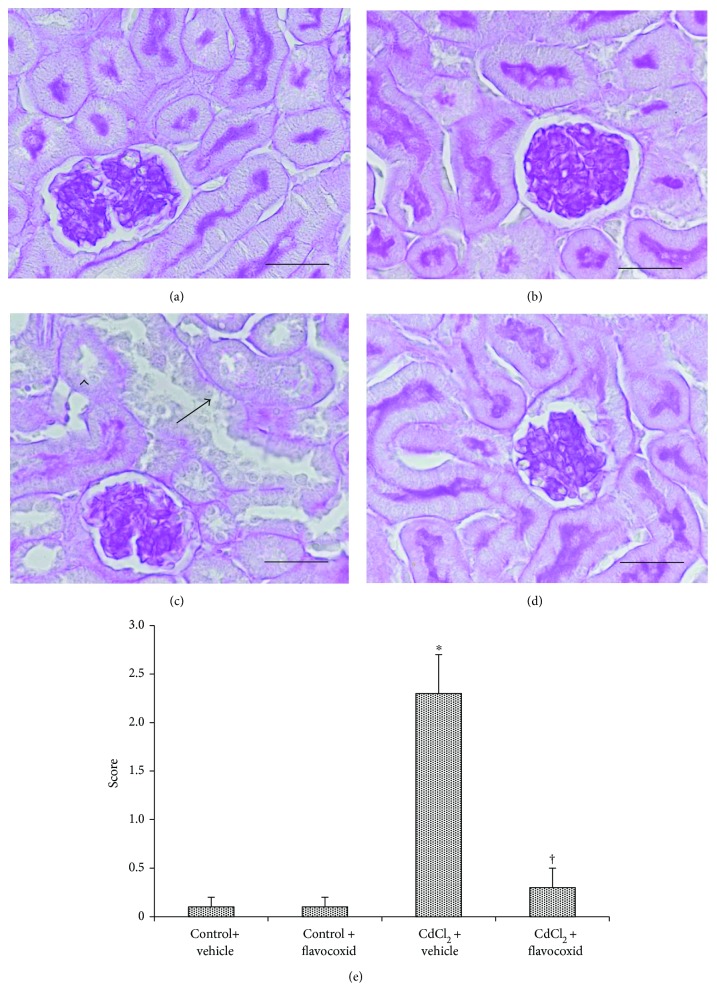
Tubular brush border of kidney sections from mice of control plus vehicle (0.9% NaCl, 1 ml/kg/day i.p.), control plus flavocoxid (20 mg/kg/day i.p.), CdCl_2_ (2 mg/kg/day i.p.) plus vehicle, and CdCl_2_ plus flavocoxid groups (periodic acid-Schiff stain). (a, b) In both control plus vehicle and control plus flavocoxid-treated mice, the proximal tubules show a regular and well-stained brush border. (c) In CdCl_2_-challenged mice, the brush border is particularly thin or absent (arrowhead) and the tubular epithelium shows structural changes (arrow), with mild interstitial edema. (d) In CdCl_2_-challenged mice administered with flavocoxid, the brush border has normal organization. (e) Tubular damage evaluation indicated by the brush border behavior. ^∗^*p* < 0.05 versus both controls and ^†^*p* < 0.05 versus CdCl_2_ plus vehicle (scale bar: 50 *μ*m).

In kidney sections stained with PAS, proximal tubules of both control groups of mice showed a regular and well-stained brush border (Figures 3(a) and 3(b)). On the contrary, in CdCl_2_-challenged mice, the thinning of the brush border with partial or total absence of the tubular epithelium was observed (Figure 3(c)). In CdCl_2_-challenged mice administered with flavocoxid, the brush border was normal (Figure 3(d)). The tubular damage evaluation demonstrated a statistically significant higher score in CdCl_2_-challenged mice and a normal score in CdCl_2_ challenged mice administered with flavocoxid (Figure 3(e)).

**Figure 4:**
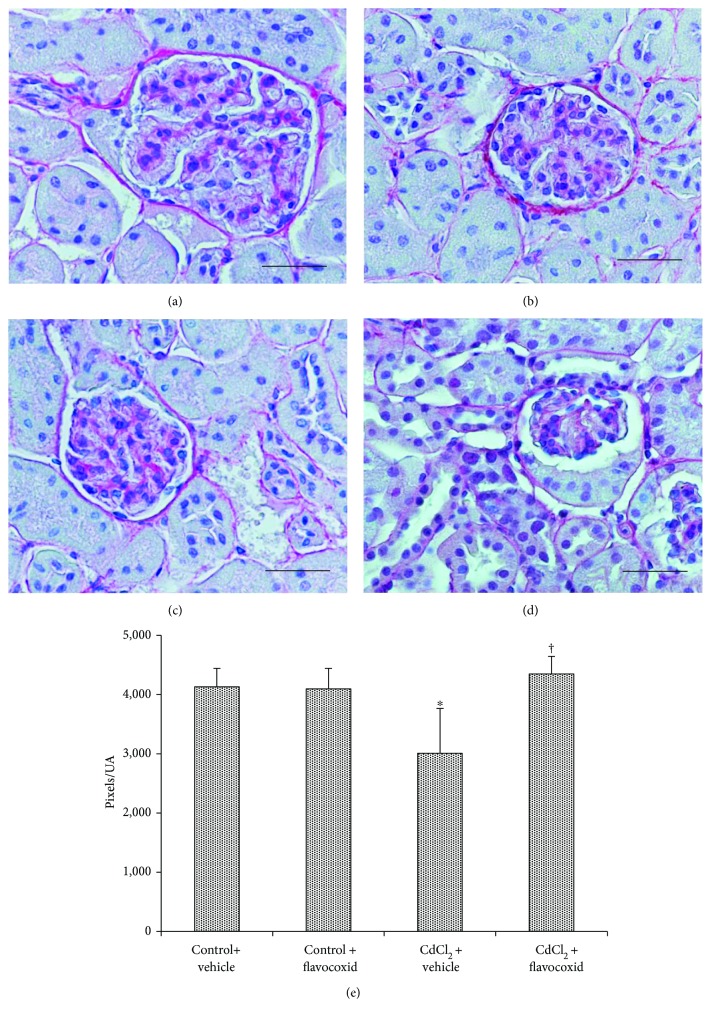
Structural organization of the interstitial connective tissue of kidneys from mice of control plus vehicle (0.9% NaCl, 1 ml/kg/day i.p.), control plus flavocoxid (20 mg/kg/day i.p.), CdCl_2_ (2 mg/kg/day i.p.) plus vehicle, and CdCl_2_ plus flavocoxid groups (Sirius red stain). (a, b) In both control plus vehicle and control plus flavocoxid-treated mice, the normal presence of collagen fibers is evident in the interstitial tissue. (c) In CdCl_2_-challenged mice, SR stain is less evident around the glomerular capsule and the tubules. (d) In CdCl_2_ plus flavocoxid-treated mice, no apparent difference with normal specimens is present. (e) Quantitative evaluation of the SR-positive areas. ^∗^*p* < 0.05 versus both controls and ^†^*p* < 0.05 versus CdCl_2_ plus vehicle (scale bar: 50 *μ*m).

In kidney sections stained with SR of both control groups of mice, glomeruli and tubules revealed a normal architecture of the collagen, which appeared formed by well-defined, red-stained fibrillary elements, standing out from the bluish-stained noncollagen components (Figures 4(a) and 4(b)). Differently, in CdCl_2_-challenged mice, SR stain was less evident around both the glomerular capsule and the tubules (Figure 4(c)). In CdCl_2_-challenged mice administered with flavocoxid, no apparent difference with normal specimens was observed (Figure 4(d)). The quantitative evaluation of the SR-positive areas showed a statistically significant decrease of the pink/red-colored collagen fibers in CdCl_2_-challenged mice, while both controls and CdCl_2_-challenged mice administered with flavocoxid demonstrated similarly not significant values (Figure 4(e)).

### 3.6. Flavocoxid Effects on Kidney Ultrastructure

**Figure 5:**
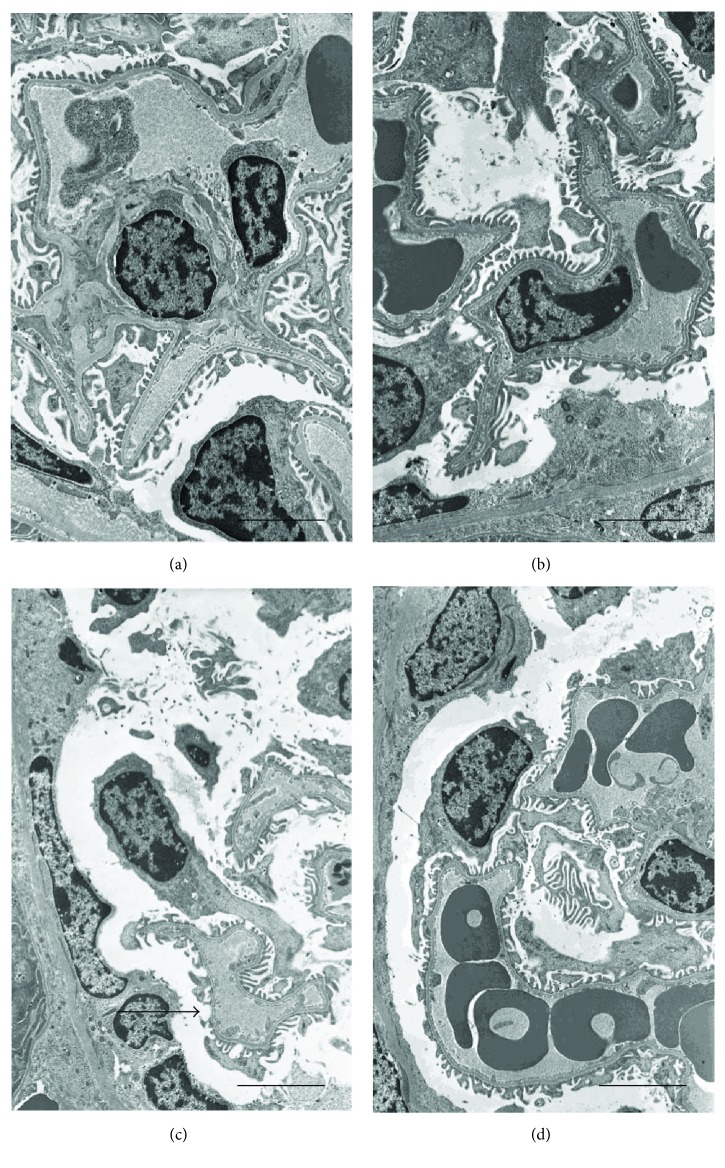
Ultrastructural organization of the glomeruli of kidneys from mice of control plus vehicle (0.9% NaCl, 1 ml/kg/day i.p.), control plus flavocoxid (20 mg/kg/day i.p.), CdCl_2_ (2 mg/kg/day i.p.) plus vehicle, and CdCl_2_ plus flavocoxid groups. (a, b) In both control plus vehicle and control plus flavocoxid-treated mice, glomeruli show normal morphology of either the podocytes or the endothelial cells. (c) In CdCl_2_-challenged mice, podocytes are elongated and fewer, and their contacts with the capillaries are lacking (arrow). (d) In CdCl_2_ plus flavocoxid-treated mice, glomerular morphology was superimposable to controls (scale bar: 4 *μ*m).

When observed with TEM, kidneys from both groups of control animals showed glomeruli with normal morphology of either the podocytes or the endothelial cells (Figures 5(a) and 5(b)). By contrast, in CdCl_2_-challenged mice, podocytes were elongated and fewer, so that their contacts with the capillaries were sometimes lacking (Figure 5(c)). In CdCl_2_-challenged mice coadministered with flavocoxid, glomerular morphology was superimposable to controls (Figure 5(d)).

**Figure 6:**
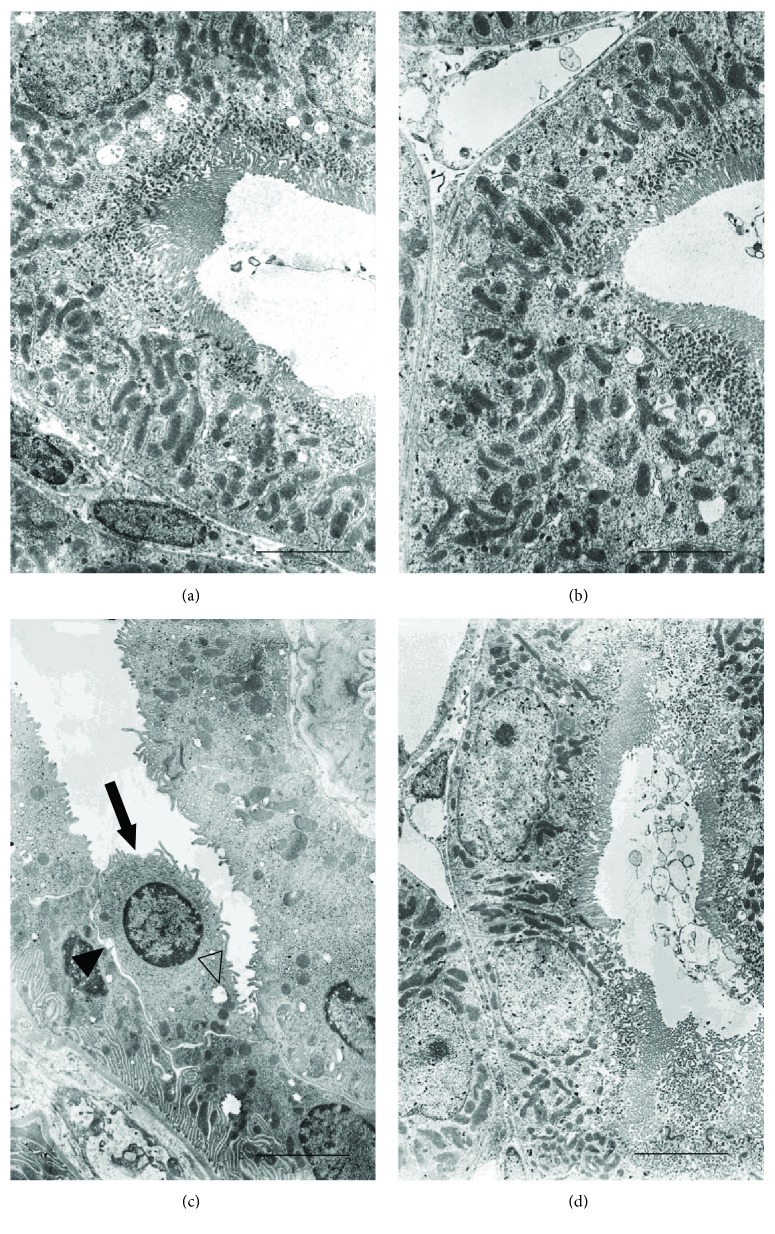
Ultrastructural organization of the proximal tubules of kidneys from mice of control plus vehicle (0.9% NaCl, 1 ml/kg/day i.p.), control plus flavocoxid (20 mg/kg/day i.p.), CdCl_2_ (2 mg/kg/day i.p.) plus vehicle, and CdCl_2_ plus flavocoxid groups. (a, b) In both control plus vehicle and control plus flavocoxid-treated mice, the epithelium of the proximal tubules shows a well-preserved brush border, normal intercellular junctions, and elongated mitochondria. (c) In CdCl_2_-challenged mice, apical microvilli are short, few, or sometimes absent (arrow), the intercellular spaces are wide (full arrowhead), and the tubular cells show round or swollen mitochondria and cytoplasmic vacuoles (empty arrowhead). (d) In CdCl_2_ plus flavocoxid-treated mice, no apparent tubular lesions are present (scale bar: 4 *μ*m).

The proximal tubules of both groups of control animals showed well-preserved microvilli of the brush border, normal intercellular junctions, and elongated mitochondria (Figures 6(a) and 6(b)). In CdCl_2_-challenged mice, the apical microvilli were shorter, fewer, or sometimes absent, the intercellular spaces were wider, and the tubular cells showed round or swollen mitochondria and cytoplasmic vacuoles (Figure 6(c)). In CdCl_2_-challenged mice administered with flavocoxid, no apparent tubular lesion was observed (Figure 6(d)).

## 4. Discussion

Free radicals have been associated in the etiology of many human diseases, among which cardiovascular and gastrointestinal disorders, cancers, neurological disorders, diabetes, ischemia/reperfusion, and ageing are included [27].

Reactive oxygen species (ROS) are products of normal cellular metabolism and may play both deleterious and beneficial roles in living systems [48]. The harmful effect of free radicals causes potential biological damage, and it is termed oxidative stress.

The kidney is a highly sensitive organ to oxidative stress, owing, in part, to its function as an oxygen sensor [49].

Cd, a serious environmental toxicant [50] found in phosphate fertilizers, in rechargeable nickel-cadmium batteries, and in tobacco is accumulated in the kidney, where its half-life was calculated over 15–30 years [51]. The proximal tubular epithelium, owing to its active role in reabsorption, is particularly sensitive to damage by oxidative stress, so that a possible link between Cd toxicity and renal cell injury was described [24]. However, Cd-induced structural changes of the glomeruli, consisting in increased mesangial matrix and glomerular swelling with wider urinary space, were also described [18, 19].

As to the mechanism involved, Cd is not a Fenton metal and it cannot produce redox reactions in biological systems. When it penetrates into the cells, Cd affects the function of many proteins interfering with the redox status of the cell, displacing endogenous redox active metals, such as iron and copper, from many cellular proteins, thus increasing the concentration of these unbound ions [20]. Furthermore, Cd may damage mitochondria inducing ROS [23], induce cellular death, in particular apoptosis [24], determine the disruption of cadherin-mediated cell-cell adhesion in the proximal tubule cells [25], and stimulate the inflammation pathways [9].

Several protective agents were found effective in defending against Cd-induced nephrotoxicity [9, 16, 19, 52–55].

In recent years, numerous studies have been performed on the potential therapeutic properties of extracts from various medicinal plants. The beneficial effects of flavonoids and natural antioxidants commonly found in vegetables, fruits, and beans were evaluated [56]: in particular, flavocoxid, a flavonoid containing a combination of extracts from *Scutellaria baicalensis* (baicalin) and *Acacia catechu* (catechin), revealed anti-inflammatory, antibacterial, antiviral, and anticancer properties and positive cardiovascular effects [35, 36]. Furthermore, the association of baicalin and catechin showed strong antioxidant activity both *in vitro* and *in vivo* [37], preventing the generation of MDA and inhibiting COX-2 and 5-LOX in mouse testes after Cd administration [38].

As no data are currently available on this topic, we investigated the effects of the coadministration of flavocoxid on Cd-induced kidney toxicity to find a new therapeutic approach based on natural antioxidants to prevent and counteract ROS generation.

CdCl_2_-treated mice showed an increased expression of iNOS in renal tissue, which could be related to the generation of ROS secondary to the structural lesions of tubular epithelial cells [26]. The consequent generation of nitric oxide may represent an important mediator of renal injury, able to induce the progression to renal failure. In fact, after CdCl_2_ administration, iNOS was significantly increased in kidneys of mice when compared to controls. Flavocoxid showed a positive action on iNOS expression, which was reduced to close to control values.

When oxidative stress occurs, the consequent overproduction of ROS overcomes the cellular defence systems and activates many signaling protein kinases and transcription regulatory factors [27]. Among signaling protein kinases, an important role is played by extracellular signal-related kinases (ERKs) 1 and 2, which are members of the mitogen-activated protein kinase (MAPK) family. pERKs show an increased expression during the onset of inflammation [57] and can regulate gene expression, cell proliferation, apoptosis, differentiation, cell-matrix interactions, and cell migration [58]. It was demonstrated that CdCl_2_ administration induces direct effects both *in vitro* and *in vivo* on specific inflammatory mediators and markers [59], among which pERKs are included [60]. As to the way by which pERK1/2 is stimulated by Cd challenge, in human endothelial cells a role of the epidermal growth factor receptor (EGFR) was proposed as the main target of ROS [61]. We have already shown that in mouse testis, p-ERK 1/2 was markedly expressed in CdCl_2_-challenged mice and flavocoxid was able to significantly reduce p-ERK 1/2 overexpression [38]. In the kidney, we demonstrated that CdCl_2_ administration leads to an activation of pERK1/2 and, more importantly, that flavocoxid counteracted the increased pERK expression.

Recently, oxidative stress has been also considered able to increase circulating MMP-9 activity in patients with resistant albuminuria [28]. MMP-9, also called gelatinase B or 92 kDa gelatinase/type IV collagenase, can be activated either *in vitro* and *in vivo* [29], and it has a great specificity for substrates such as different types of collagen, proteoglycans, and elastin [29]. A higher expression of MMP-9 was shown in transformed PDV cell lines through the ROS-NF*κ*B mechanism [62]. Furthermore, in human endothelial cells [61] and in embryonic BNL CL2 cells [29], MMP-9 levels were increased after exposure to Cd: the role of intracellular ROS was demonstrated through the activation of the EGFR, NF*κ*B, and activator protein-1 pathways.

As Cd exposure causes oxidative stress, MMP-9 activity was evaluated in kidneys of mice exposed to CdCl_2_. It was shown that MMP-9 expression was significantly increased after 14 days of CdCl_2_ administration, while it was significantly reduced after treatment with flavocoxid, thus indicating that a relationship exists also in the kidney between Cd exposure and MMP-9 expression, most likely secondary to oxidative stress.

As a consequence of the higher MMP-9 expression, the presence of collagen was reduced in CdCl_2_-challenged mice around both the glomerular capsule and the tubules in SR-stained specimens. MMP-9 specifically cleaves type IV collagen, which can degrade most components of the basal membrane either in embryonic development or in *in vitro* models [63]. Therefore, the increased MMP-9 levels could be responsible for tissue collagen-reduced stain at the basement membrane level of both glomeruli and tubules demonstrated with SR stain. These morphological data seem to be in contrast with previous results from a stereological study on mouse kidney exposed for four weeks to CdCl_2_ [19], where an increased volume of the fibrous tissue was observed in HE-stained specimens compared to controls. The differences could be related to the longer time of exposure to Cd in the work of Rafati et al. [19], which could have induced a more serious hypoxia with increase in fibrosis [64].

As to the structural organization of the kidneys of mice challenged with CdCl_2_ alone or treated with flavocoxid, the behavior of glomeruli and proximal tubules was evaluated.

When glomeruli were considered, their total area was significantly increased in kidneys of mice exposed to CdCl_2_ when compared to the controls. On the contrary, in CdCl_2_-challenged mice coadministered with flavocoxid, the glomerular area was significantly reduced if compared to the CdCl_2_-alone group. These data indicated that glomerular swelling, expression of kidney pathology [65] occurring in many renal diseases such as diabetes [44, 45], and cardiorenal syndrome [53] were present also after Cd exposure, as previously demonstrated [19].

Transmission electron microscopic examination showed ultrastructural changes in glomeruli of CdCl_2_-challenged animals. Previous studies demonstrated that in Cd-challenged rats, glomeruli exhibited thickening of the basement membrane, irregular foot processes, and myelin figures in podocytes [16]. In this study, Cd-induced alterations in glomeruli, in addition to the previously discussed increase of their area, included podocytes with elongated, swollen, and sometimes absent pedicles, thus indicating a negative role of Cd in the filtration system of the kidney. We demonstrated, as far as we know, for the first time that the antioxidant treatment with flavocoxid reduced glomerular damage, as supported by the morphological data and by the morphometric analysis.

As to the cells of the proximal tubule, in CdCl_2_-challenged mouse lysosomes and myelin bodies, distorted or ring-shaped mitochondria, cytoplasmic vacuolization or cytolysis [13], decreased, irregular microvilli [16], fragmented and short basolateral invaginations [15], and condensed chromatin [17] have been described.

Our TEM micrographs showed that the proximal tubules had wide intercellular spaces and peculiar changes in their cells, such as round or swollen mitochondria, cytoplasmic vacuoles, and short, few, or sometimes absent apical microvilli. This morphological aspect in CdCl_2_-challenged mice was also confirmed by the PAS-stained sections. In fact, the PAS reaction stain structures correlated with adhesiveness and tightness of membranes, such as brush borders of tubules and basement membranes. Its lower positivity, or even its negativity, could reflect serious impairment of reabsorptive processes of the tubules, or even the absence of the mechanisms of active transport [14].

On the contrary, we demonstrated that flavocoxid protects against the CdCl_2_-induced structural and ultrastructural alterations in kidney of mice, as indicated by the organization of the proximal tubules. In fact, in CdCl_2_-challenged mice coadministered with flavocoxid, the PAS reaction was close to normal, the brush border was preserved, and the tubular damage score, higher in CdCl_2_-challenged mice, was also normal.

This can be attributed to the antioxidant and free radical quenching efficacy of flavocoxid, which significantly reduced the oxidative stress [38].

## 5. Conclusion

**Figure 7:**
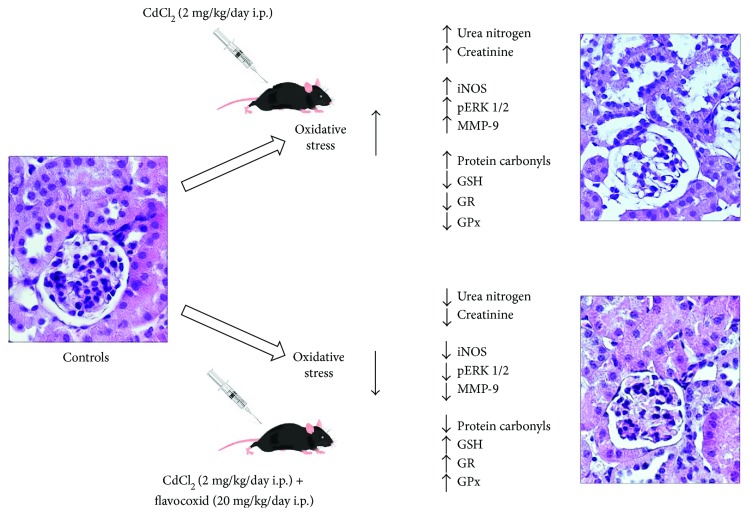
Graphical model indicating the different effects of CdCl_2_ alone and CdCl_2_ plus flavocoxid on the mouse kidney. CdCl_2_ = cadmium chloride; iNOS = inducible nitric oxide synthase; pERK 1/2 = phosphorylated extracellular signal-regulated protein kinase 1/2; MMP-9 = matrix metalloproteinase 9; GSH = reduced glutathione; GR = glutathione reductase; GPx = glutathione peroxidase.

The present study suggests that flavocoxid was able to significantly reduce CdCl_2_-induced oxidative damage secondary to ROS generation in the kidney (Figure 7). In fact, flavocoxid significantly lowered iNOS, pERK 1/2, and MMP-9 expression and reduced morphological changes of glomeruli and proximal tubules, which are known as key renal targets for Cd. The use of flavocoxid, a natural antioxidant, can be included among the several experimental strategies, which may have beneficial effects on kidney in humans during or after heavy metal exposure.

## Figures and Tables

**Table 1:** Urea nitrogen and creatinine levels in mice exposed to cadmium chloride (CdCl_2_; 2 mg/kg i.p.) plus vehicle, as compared to mice exposed to CdCl_2_ (2 mg/kg i.p.) plus flavocoxid (20 mg/kg/day i.p.) or to control mice treated with vehicle or flavocoxid alone.

	Urea nitrogen (mg/dl)	Creatinine (mg/dl)
Control + vehicle	13.6 ± 1.3	0.58 ± 0.04
Control + flavocoxid	13.9 ± 1.4	0.61 ± 0.08
CdCl_2_ + vehicle	39.3 ± 4.4^a^	1.48 ± 0.18^a^
CdCl_2_ + flavocoxid	17.5 ± 2.2^b^	0.71 ± 0.09^b^

All the values are expressed as mean ± SE, *n* = 7 animals for each group. ^a^*p* < 0.05 versus both controls and ^b^*p* < 0.05 versus CdCl_2_ + vehicle.

**Table 2:** Protein carbonyl levels and GSH content in mice exposed to cadmium chloride (CdCl_2_; 2 mg/kg i.p.) plus vehicle, as compared to mice exposed to CdCl_2_ (2 mg/kg i.p.) plus flavocoxid (20 mg/kg/day i.p.) or to control mice treated with vehicle or flavocoxid alone.

	Protein carbonyls (*μ*mol/mg protein)	GSH (*μ*mol/g tissue)
Control + vehicle	0.004 ± 0.001	70 ± 3
Control + flavocoxid	0.005 ± 0.001	72 ± 4
CdCl_2_ + vehicle	0.009 ± 0.002^a^	52 ± 6^a^
CdCl_2_ + flavocoxid	0.006 ± 0.001^b^	66 ± 5^b^

All the values are expressed as mean ± SE, *n* = 7 animals for each group. ^a^*p* < 0.05 versus both controls; ^b^*p* < 0.05 versus CdCl_2_ + vehicle.

**Table 3:** Glutathione reductase (GR) and glutathione peroxidase (GPx) levels in mice exposed to cadmium chloride (CdCl_2_; 2 mg/kg i.p.) plus vehicle, as compared to mice exposed to CdCl_2_ (2 mg/kg i.p.) plus flavocoxid (20 mg/kg/day i.p.) or to control mice treated with vehicle or flavocoxid alone.

	GR (nmol/min per mg protein)	GPx (nmol/min per mg protein)
Control + vehicle	20.03 ± 1.12	33.42 ± 1.78
Control + flavocoxid	19.83 ± 1.04	31.64 ± 1.83
CdCl_2_ + vehicle	12.64 ± 0.63^a^	18.72 ± 0.87^a^
CdCl_2_ + flavocoxid	18.35 ± 1.01^b^	28.69 ± 1.53^b^

All the values are expressed as mean ± SE, *n* = 7 animals for each group. ^a^*p* < 0.05 versus both controls and ^b^*p* < 0.05 versus CdCl_2_ + vehicle.

## Abbreviations

CdCl_2_: Cadmium chloride
ROS: Reactive oxygen species
iNOS: Inducible nitric oxide synthase
pERK 1/2: Phosphorylated extracellular signal-regulated protein kinase 1/2
MMP-9: Matrix metalloproteinase 9
HE: Hematoxylin and eosin
PAS: Periodic acid-Schiff
SR: Sirius red
MDA: Malondialdehyde
COX: Cyclooxygenase
LOX: Lipooxygenase
MAPK: Mitogen-activated protein kinase
PCO: Protein carbonyl content
GSH: Glutathione
GR: Glutathione reductase
GPx: Glutathione peroxidase.
